# IL-10-Mediated Refueling of Exhausted T Cell Mitochondria Boosts Anti-Tumour Immunity

**DOI:** 10.20900/immunometab20210030

**Published:** 2021-09-24

**Authors:** Dylan Ryan, Christian Frezza

**Affiliations:** MRC Cancer Unit, University of Cambridge, Cambridge Biomedical Campus, Cambridge CB2 0XZ, UK

**Keywords:** immunotherapy, mitochondria, metabolism, T cell, exhaustion, IL-10

## Abstract

Immunotherapy has underscored a revolution in cancer treatment. Yet, many patients fail to respond due to T cell exhaustion. Here, an intervention that restores mitochondrial function reversed the exhausted T cell phenotype to promote cytotoxicity and durable anti-tumour responses in vivo.

Immunotherapy is a type of biological therapy that uses the bodys own immune system to treat cancer. It is often achieved by adoptive T cell transfer (ACT) and immune checkpoint blockades (ICBs), which preferentially target tumour-infiltrating lymphocytes (TILs), but fails to work in most patients. This failure is often attributed to the adoption of an “exhausted” T cell phenotype. Defining T cell exhaustion is complex, but the term primarily describes CD8^+^ effector T cells with a reduced capacity to secrete cytokines and elevated expression levels of inhibitory receptors [1]. In tumours, exhaustion of TILs can impair cytotoxic killing, which subsequently enables tumour progression. Two major subsets of exhausted CD8^+^ T cells include an ICB-responsive “progenitor exhausted” subset that can proliferate and differentiate into an ICB-unresponsive “terminally exhausted”. Nutrient restriction in the tumour microenvironment (TME) and impaired mitochondrial bioenergetics of TILs is a contributing factor to exhaustion and impaired anti-tumour killing. Writing in *Nature Immunology*, Guo et al. explored the possibility of reinvigorating terminally exhausted CD8^+^ T cells by combining a “metabolic intervention”, comprised of an IL-10-Fc fusion protein to restore mitochondrial fitness, in combination with ACT or ICB immunotherapy [2]. Their findings provide strong preclinical evidence that metabolic interventions can promote proliferation and cytotoxic activity of terminally exhausted effector T cells to enhance cancer immunotherapy.

Interleukin-10 (IL-10) is a cytokine that can promote anti-tumor immunity. In this work, the authors first synthesized a recombinant fusion protein between human IL-10 and IgG1 (IL-10-Fc), which would cross-react with the mouse IL-10 receptor and extend the half-life of the protein. To test the ability of IL-10-Fc to regulate T cell responses in vivo, the authors employed the poorly immunogenic B16F10 melanoma mouse model. They found that ACT of tumour antigen-specific PMEL CD8^+^ T cells with IL-10-Fc enhanced tumour infiltration of TILs and increased the number of CD8^+^ T cells with minimal impact on other lymphocytes or myeloid cells. Importantly, subset analysis revealed a specific expansion and improved effector functioning of terminally exhausted CD8^+^ T cells (PD1^+^TIM-3^+^ CD8^+^ T cells) in an IL-10 receptor-dependent manner. To confirm a direct effect of IL-10-Fc on terminally exhausted CD8^+^ T cells, independent of progenitor exhausted T cells, the authors first demonstrated an antigen-specific increase in PD1^+^TIM-3^+^-double positive populations when they were co-administered with OT-I CD8^+^ T cells. Likewise, comparing the transfer of PD1^+^TIM-3^-^CD8^+^ (progenitor exhausted) and PD1^+^TIM-3^+^CD8^+^ (terminally exhausted) T cells confirmed a specific expansion of terminally exhausted T cells even in the absence of progenitor exhausted T cells. Finally, in vivo depletion of progenitors using diphtheria toxin (DT) and *Tcf7* ^DTR-GFP^ transgenic P14 T cells had no impact on the expansion and tumour-killing capacity of terminally exhausted T cells in B16-gp33 tumours. These results show that IL-10-Fc can directly reinvigorate terminally exhausted CD8^+^ T cells and promote tumour killing via a progenitor-independent mechanism.

IL-10 signaling is reported to oppose pro-inflammatory macrophage activation by antagonizing mTOR and promoting mitophagy [3]. Given the profound effect of IL-10 on macrophage metabolic reprogramming, the authors examined whether IL-10-Fc could elicit effects on the respiratory activity of CD8^+^ T cells. Using in vitro co-cultures of activated PMEL CD8^+^ T cells and B16F10 cancer cells, and ex vivo production of PD1^+^TIM-3^+^CD8^+^ T cells, respirometry analysis revealed an increase in oxygen consumption rates (OCR) upon treatment with IL-10-Fc. The increase in OCR was also accompanied by increased PMEL CD8^+^ T cell proliferation and cytotoxicity against B16F10 cells and was dependent on the IL-10 receptor. To determine whether the metabolic response was conserved between mouse and human T cells, the authors validated the ability of IL-10-Fc to increase respiration, proliferation and the cytotoxic activity of CAR-T cells targeting HER2-expressing cancer cells. These results clearly demonstrated that IL-10-Fc could promote oxidative phosphorylation (OXPHOS) of mouse and human T cells and suggested that IL-10-Fc-induced metabolic reprogramming may underlie the enhanced proliferation and killing capacity in vitro.

To explore the utility of using IL-10-Fc in vivo as an adjunct to cancer immunotherapy, Guo et al. utilized four different solid tumour models to assess safety and efficacy. Firstly, IL-10-Fc was shown to reduce B16F10 tumour burden on its own and to synergize with ACT of PMEL CD8^+^ T cells. Secondly, ACT of OT-I CD8^+^ T cells with IL-10-Fc led to potent tumour regression in a large and established YUMM1.7-OVA melanoma model. Thirdly, a combination of HER2-targeting CAR-T cells led to a 90% reduction in tumour size using the HER2-expressing MC38 model of colon adenocarcinoma. This finding was particularly notable as no pre-conditioning was required for the robust anti-tumour activity of CAR-T cells. Overall, combination treatment of IL-10-Fc with ACT led to greater survival rates, and remarkably, sustained anti-tumour immune memory with many IL-10-Fc-treated mice surviving tumour re-challenge. Lastly, IL-10-Fc was also found to synergize with anti-PD1 ICB immunotherapy in a mouse CT26 colorectal tumour model leading to tumour eradication and durable immune responses. These preclinical studies suggest that IL-10-Fc may successfully potentiate ACT and ICB immunotherapies with no overt toxicity.

To mechanistically dissect how IL-10-Fc reinvigorated terminally exhausted CD8^+^ T cells in vivo, RNA sequencing (RNA-seq) analysis of PD1^+^TIM-3^+^CD8^+^ TILs from B16F10 tumours treated with either PBS or IL-10-Fc was performed. Enrichment analysis revealed increases in genes relating to OXPHOS, effector T cell responses and a decrease in inhibitory receptor expression. PD1^+^TIM-3^+^CD8^+^ TILs isolated from tumours were also found to have elevated basal OCR and mitochondrial reactive oxygen species (mtROS) levels. To uncover a causal relationship between metabolic reprogramming and T cell reinvigoration, the authors took a pharmacological approach targeting key bioenergetic pathways, including glycolysis, glutaminolysis, and fatty acid oxidation (FAO). Here, the authors found an important role for glycolysis-derived pyruvate uptake into mitochondria via the mitochondrial pyruvate carrier (MPC) for the increase in IL-10-Fc-driven respiration and validated this using MPC-KO T cells. Furthermore, feeding OT-I CD8^+^ T cells with sodium pyruvate circumvented the need for IL-10-Fc and promoted PD1^+^TIM-3^+^CD8^+^ T cell proliferation. IL-10-Fc failed to promote expansion and anti-tumour cytotoxic activity of terminally exhausted CD8^+^ T cells in MPC-KO OT-I CD8^+^ T cells in both B16F10-OVA tumours and YUMM1.7-OVA tumours. These findings demonstrate that IL-10-Fc promotes OXPHOS and anti-tumour killing via a pyruvate and MPC-dependent refueling of mitochondria (Figure 1).

While the involvement of mitochondrial pyruvate uptake and OXPHOS in reinvigorating terminally exhausted T cells is evident, precisely how IL-10-Fc regulates MPC-dependent OXPHOS remains to be established. Given the known role of IL10-Fc in promoting mitophagy of damaged mitochondria [3], perhaps a similar mechanism is at play in terminally exhausted CD8^+^ T cells. In addition, improved mitochondrial fitness achieved by enforced peroxisome proliferator-activated receptor-gamma coactivator (PGC)--1α overexpression (and subsequently increased mitochondrial biogenesis) in CD8^+^ TILs improved anti-tumour efficacy in a mouse model of melanoma [4]. This work highlights the need to develop more therapeutic approaches that enhance mitochondrial fitness to improve T cell function and patient responses to immunotherapy. Since pyruvate alone was sufficient to re-activate mitochondrial functions in exhausted CD8^+^ T cells, it is tempting to speculate that other dietary interventions could have similar effects, paving the way for diet-based immunomodulation. Whilst this work presents a very exciting development and compelling case for the combination of metabolic interventions and immunotherapies, it remains to be seen if these results will be applicable to a broad range of cancer types. The relevance of these findings to the clinic is also unclear as these experiment use highly immunogenic tumour antigens and involved a peritumoral mode of delivery for IL-10-Fc. Finally, a large gap still remains in the translation of preclinical mouse model therapies to the effective treatment of human disease.

## Figures and Tables

**Figure 1:**
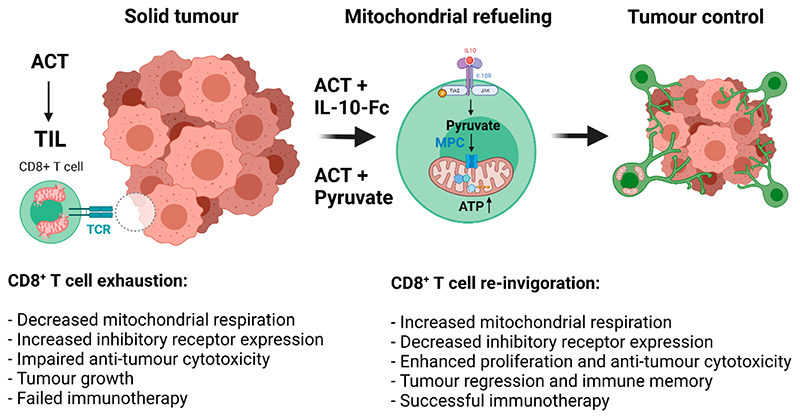
IL-10-Mediated Refueling of Exhausted T Cell Mitochondria Boosts Anti-Tumour Immunity. Figure created with Biorender.com.
